# Apparent diffusion coefficient of hepatocellular carcinoma on diffusion-weighted imaging: Histopathologic tumor grade versus arterial vascularity during dynamic magnetic resonance imaging

**DOI:** 10.1371/journal.pone.0197070

**Published:** 2018-05-11

**Authors:** In Kyung Park, Jeong-Sik Yu, Eun-Suk Cho, Joo Hee Kim, Jae-Joon Chung

**Affiliations:** Department of Radiology, Gangnam Severance Hospital, Yonsei University College of Medicine, Gangnam-Gu, Seoul, Korea; Universita degli Studi di Pisa, ITALY

## Abstract

**Objectives:**

Apparent diffusion coefficient (ADC) has been suggested to reflect the tumor grades of hepatocellular carcinomas (HCCs); *i*.*e*., it can be used as a biomarker to predict the patients’ prognosis. To verify its feasibility as a biomarker, the present study sought to determine how the ADC values of HCC are affected by a tumor’s histopathologic grade and arterial vascularity.

**Materials and methods:**

From 131 consecutive patients, 141 surgically resected HCCs (16 well-differentiated [wd-HCCs], 83 moderately-differentiated [md-HCCs], and 42 poorly-differentiated HCCs [pd-HCCs]) were subjected to a comparison of the tumors’ arterial vascularity (non-, slightly-, or markedly-hypervascular) determined on dynamic magnetic resonance imaging (MRI) and the ADC was measured retrospectively.

**Results:**

The pd-HCCs (1.05±0.16 × 10^−3^ mm^2^/s) had a significantly lower ADC than md-HCCs (1.16±0.21 × 10^−3^ mm^2^/s; p = 0.010), but there was no significant difference compared to wd-HCCs (1.11±0.18 × 10^−3^ mm^2^/s; p = 0.968). The mean ADC was significantly higher in markedly hypervascular lesions (1.20±0.20 × 10^−3^ mm^2^/s) than in nonhypervascular lesions (0.95±0.14 × 10^−3^mm^2^/s; p<0.001) or slightly hypervascular lesions (1.04±0.15 × 10^−3^mm^2^/s; p<0.001). The ADC values and arterial vascularity were significantly correlated in wd-HCCs (p = 0.005) and md-HCCs (*p*<0.001). The mean ADC of pd-HCCs was significantly lower than those of other lesions, even in the markedly hypervascular lesion subgroup (p = 0.020).

**Conclusion:**

Although pd-HCC constantly shows low ADCs regardless of arterial vascularities, ADCs cannot stably stratify histopathologic tumor grades due to the variable features of wd-HCCs; and the ADC should be used with caution as a tumor biomarker of HCC.

## Introduction

Despite improvements in diagnosis and patient management, hepatocellular carcinoma (HCC) is the second leading cause of cancer-related deaths [1]. Poorly-differentiated (pd)-HCC have a poor prognosis because of its higher recurrence rate compared to well-differentiated (wd)-HCC and moderately-differentiated (md)-HCC [2–4]. The 5-year recurrence rate of pd-HCC is up to 75% [5] after surgical resection and 8–20% after liver transplantation [6]. Preoperative prediction of histopathologic differentiation for HCC is helpful for optimal treatment planning [7, 8].

Diffusion-weighted imaging (DWI) with apparent diffusion coefficients (ADCs) obtained from DWI data has been introduced in abdominal imaging and is becoming a standard protocol of diagnostic magnetic resonance imaging (MRI) of the liver. This technique provides visualization of random Brownian motion of water molecules [9, 10]. The use of DWI initially focused on the detection of tiny liver metastases by its synergistic effect with T2-weighted imaging and dynamic enhanced imaging [11–16]. It is also a promising technique for tissue characterization, based on the diffusional properties of water molecules through biologic tissues [17]. Some studies have demonstrated that DWI aids in distinguishing early HCCs from benign regenerative nodules in patients with chronic liver disease [18, 19]. Moreover, DWI has been suggested to predict the histopathologic grade of malignant hepatic tumors because of an inverse correlation between ADC values and tumor grades [20–27]. However, in clinical practice, we have encountered exceptional cases of early lesions of hepatocarcinogenesis showing low ADCs. No consensus has been reached on this issue. The ADC inherently contains the perfusion fraction, which could be affected by tumor vascularity; hence, the contribution of tumor enhancement cannot be excluded in determining the ADC of HCCs. To verify its feasibility as a biomarker, the present study sought to determine how the ADC values of HCC are affected by a tumor’s histopathologic grade and arterial vascularity.

## Materials and methods

This retrospective study was approved by the Gangnam Severance Hospital institutional review board (IRB) for clinical studies, and the requirement for informed patient consent was waived.

### Patients and clinical data collection

**Fig 1:**
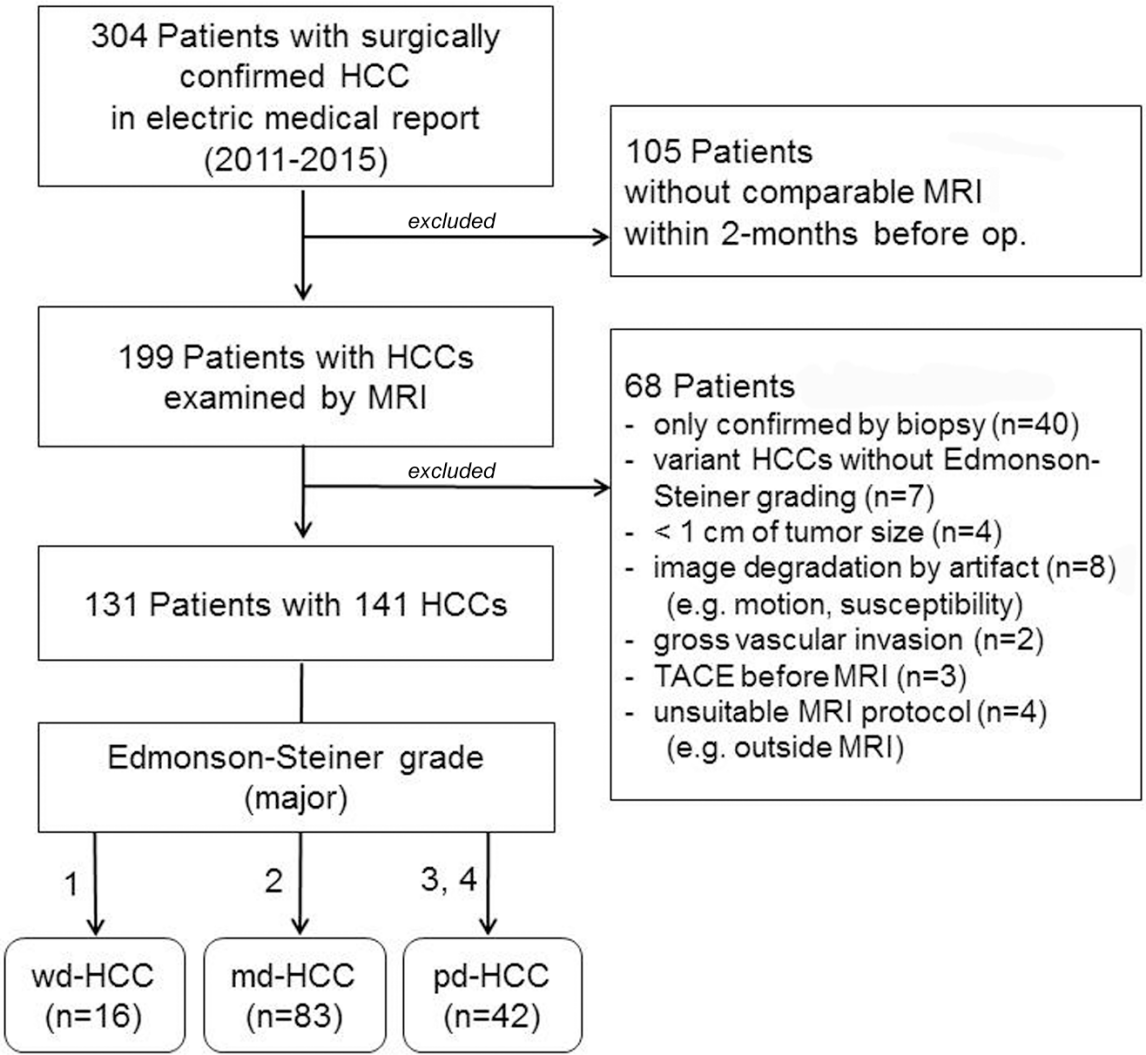
Flowchart of patient selection. HCC, hepatocellular carcinoma; wd, well-differentiated; md, moderately-differentiated; pd, poorly-differentiated; TACE, transcatheter arterial chemoembolization.

We searched the electronic medical records and radiological computer records of all patients surgically diagnosed with HCC at our institution between January 2011 and December 2015, and found 304 potential candidates. The inclusion criteria were as follows: underwent preoperative liver MRI (including dynamic enhanced imaging and DWI) within 2 months before surgery; location of tumor on MRI was the same as the surgically resected HCC in the pathologic report; the pathologic report described the histopathologic grade, based on the Edmonson and Steiner grading system; quality of dynamic enhanced imaging allowed evaluation of arterial vascularity; and definite localization of the lesion on DWI made it possible to draw regions of interest (ROIs). The exclusion criteria were as follows: lesion size was < 1 cm (to avoid the partial volume averaging effect); image was degraded because of cardiac motion or susceptibility artifact around the area of the lesions; evaluation of tumor’s vascularity was difficult due to gross vascular invasion; or tumor was treated with transcatheter arterial chemoembolization or radiofrequency ablation before imaging. From 131 patients ranging in age from 38 to 81 years (mean age, 58.1 years, men:women, 104:27), 141 lesions were finally included (Fig 1). Most patients had underlying liver disease: chronic hepatitis B (n = 108), hepatitis C (n = 9), or hepatitis B and C with or without cirrhosis (n = 1) and alcoholic cirrhosis (n = 2), while the other 11 patients had no known underlying liver disease.

### MRI protocol

We used a 1.5-T MRI system (Magnetom Avanto, Siemens, Erlangen, Germany) fitted with high-performance gradients (maximum amplitude 45 mT/m) and a six-element phased-array surface coil. After the non-contrast fast T2- and T1-weighted MRI with use of half-Fourier single-shot turbo-spin-echo and double-echo chemical shift gradient echo sequences, dynamic MRI was performed. A fat-suppressed three-dimensional (3D) gradient echo sequence (*i*.*e*., volumetric interpolated breath-hold examination [VIBE]; Siemens, Erlangen, Germany) was obtained before injecting the intravenous contrast media of gadoxetic acid (Primovist, Bayer Schering; 0.025 mmol/kg). After a 1-mL test-bolus injection of gadoxetic acid to determine the timing of the earliest phase, the contrast media was injected through a power injector at a speed of 1 mL/s, followed by a 20-mL physiological saline flush at the same rate. Dynamic imaging, including the early arterial phase, late arterial phase, and portal venous phase, was performed at 34-s intervals (20 s for image acquisition with breath-holding and 14 s for rebreathing). Corresponding subtraction arterial-dominant phase images were acquired with coregistration software (Inline Liver registration; Siemens Medical Solutions, Erlangen, Germany), which performed image-by-image subtraction of the precontrast images from the coregistered postcontrast images.

Respiratory-triggered DWI with single-shot echo planar imaging was then acquired with motion-probing gradients in three directions. Respiration was monitored using the prospective acquisition correction technique, which periodically evaluates diaphragmatic position by navigator echoes. The scanning parameters for DWI were two *b* factors, 50 s/mm^2^ and 800 s/mm^2^; TR, 3900 ms; TE, 75 ms; matrix size, 156×192; average, 6; 54–60 slices (27–30 for each *b* factor); slice thickness, 6 mm; and interslice gap, 20%. The MRI system automatically calculated the ADC values for each DWI sequence and generated the corresponding ADC maps. The delayed hepatobiliary phase with the VIBE sequence was obtained 20 min after contrast injection.

### Data analysis

The study coordinator (an abdominal radiologist with 20 years of experience) preliminarily reviewed all images, pathologic reports, and medical records. Each HCC was marked with an arrow on the gadoxetic acid-enhanced hepatobiliary phase images. For the precontrast T1-weighted images and arterial-dominant (early or late arterial) phase images combined with the corresponding subtraction images, two radiologists (a radiologist with 10 years of experience in abdominal MRI and a third-year resident) were blinded to the histologic grade of the lesions. They independently determined the arterial vascularity of each tumor using a three-point scoring system: a score of 1 (*i*.*e*., nonhypervascular) indicated a hypointense lesion on arterial-dominant phase images, regardless of the findings of the subtraction images, and a hypointense or isointense lesion on the corresponding subtraction images for hyperintense lesions on precontrast T1-weighted images; a score of 2 (*i*.*e*., slightly hypervascular) indicated an isointense or slightly hyperintense lesion on arterial-dominant phase images that was a hyperintense lesion on the corresponding subtraction images; and a score of 3 (*i*.*e*., markedly hypervascular) indicated a lesion that was vividly hyperintense on arterial-dominant phase images and the corresponding subtraction images. When the two radiologists disagreed on the degree of tumor enhancement, two reviewers together reviewed the images and re-evaluated the vascularity for future analysis.

The ADC of each tumor was measured independently by the same two radiologists by using an oval or polygonal ROI on the ADC map. The largest ROI was placed on the solid portion where the previously assessed vascularity was determined on dynamic imaging. For nonhypervascular lesions, the ROI was placed on the non-necrotic solid portion by referring to other sequence images, including the precontrast T1- and T2-weighted images and DWI. The tumor’s margin was excluded to minimize the partial volume average effect. When the lesion was small and indistinguishable from surrounding hepatic parenchyma on the ADC map, the corresponding DWI images (*b* value, 50 s/mm^2^) were simultaneously displayed on picture archiving and communication system monitors. Two horizontal and perpendicular lines passing the center of the lesion were drawn from the left border and the upper border of the image to determine the *x* and *y* coordinates of the lesion on the DWI image. Two identical lines were drawn on the corresponding ADC map. The ROI was placed around the manually synchronized center of the lesion to measure the ADC values. The average values obtained by two radiologists were used for further analyses.

The pathologists reported the major and worst Edmonson and Steiner grades of HCCs for all lesions in the patients’ medical records. In the present study, the major grade was regarded to represent the imaging characteristics of each lesion. All lesions were divided into three histopathologic groups for future analysis: 16 wd-HCCs for Edmonson and Steiner grade 1 lesions; 83 md- HCCs for grade 2 lesions; 42 pd-HCCs comprising grade 3 (35 lesions) and grade 4 (7 lesions).

### Statistical analysis

Interobserver agreement regarding the degree of arterial phase contrast-enhancement was assessed using linear-weighted Cohen kappa tests. A kappa value of 0.00–0.20 indicated slight agreement; 0.21–0.40, fair agreement; 0.41–0.60, moderate agreement; 0.61–0.80, good agreement; and 0.81–1.00, excellent agreement. The Bland–Altman test was performed for the reproducibility of ADC measurement between the two observers. To ensure that the number of HCC in each group was sufficient to draw conclusion, ANOVA post-hoc power calculation was carried out using Power Analysis and Sample Size 12 for Windows software package (NCSS Inc, LLC, Kaysville, UT, USA). After using the Kolmogorov–Smirnov test to define the data distribution pattern of the measured ADC values, the relationships between the mean ADCs and two factors (*i*.*e*., the histopathologic grades and the arterial vascularity) were stratified using analysis of variance (ANOVA) tests. To verify the relationship of ADCs in each subgroup of histopathologic tumor grade for arterial vascularity and vice versa, ANOVA with Bonferroni corrected post-hoc analysis was performed. All statistical analyses were performed using SPSS Statistics version 23 (IBM Corp., Armonk, NY). Statistical significance was set at *P* < 0.05.

## Results

### Interobserver agreement and data distribution

**Fig 2:**
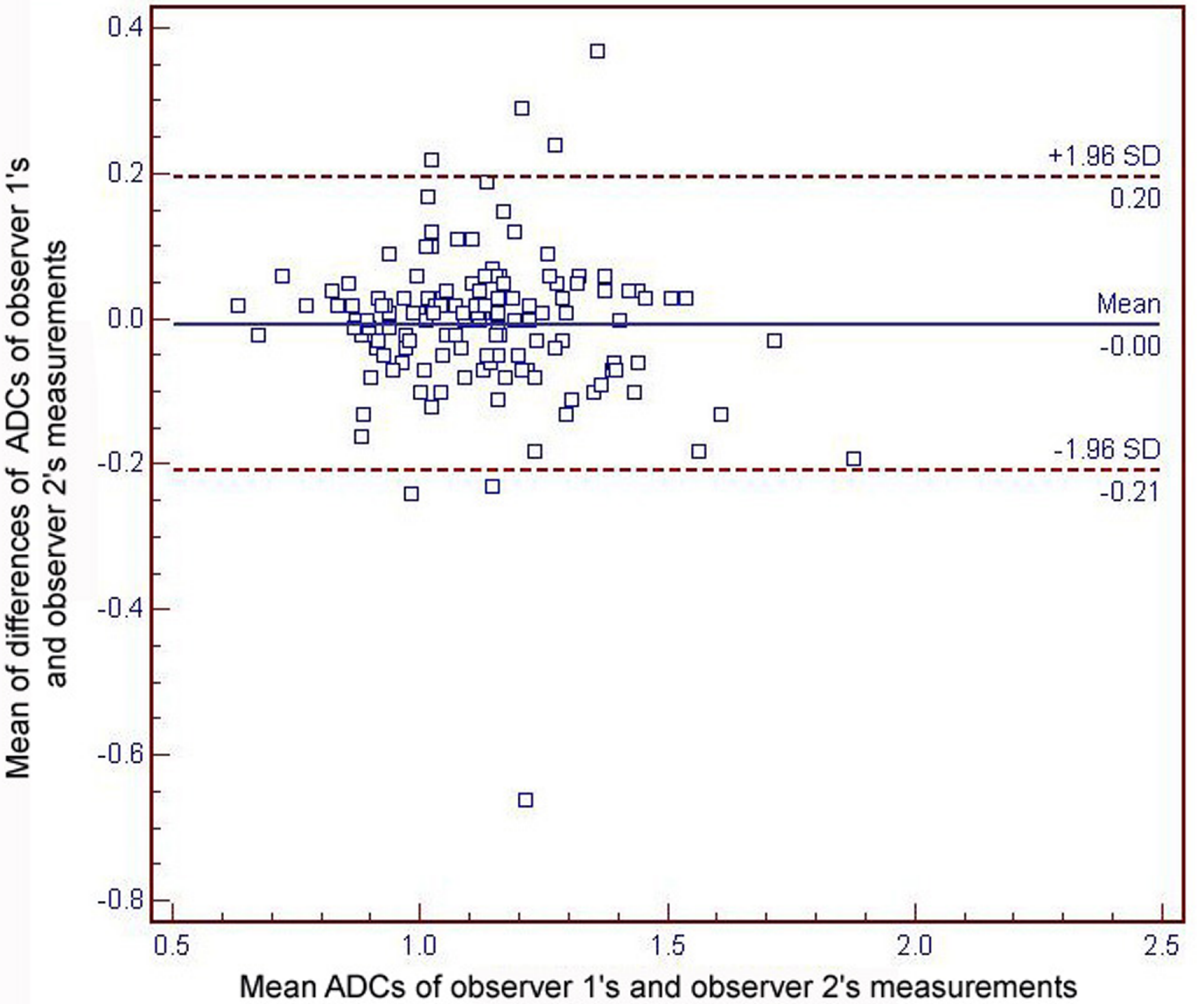
Bland–Altman plots for the reproducibility of apparent diffusion coefficients (ADCs) values between the two observers’ measurements. The blue line indicates the absolute difference and the dotted red lines indicate the 95% confidence interval of the mean difference. The mean absolute difference in the ADC measurements between the two observers is 0.004 × 10^−3^ mm^2^/s (limit of agreement, 0.20–0.66).

The Cohen kappa test revealed good interobserver agreement in the degree of arterial phase contrast-enhancement (mean kappa value = 0.770). The Bland–Altman plot (Fig 2) showed no fixed or proportional bias in the ADC assessment. In ANOVA post-hoc power calculation, the power was 82.59% between wd-HCCs, md-HCCs and pd-HCCs. Substantial reproducibility of ADC measurement between the two observers was proven. The mean absolute difference in the ADC measurement between the two observers was 0.004 × 10^−3^ mm^2^/s (limit of agreement, 0.20–0.66). There was no null distribution (*P* > 0.05). The overall ADC data showed sufficient normality to fit the subsequent statistical analyses.

### ADC and tumor grades

**Fig 3:**
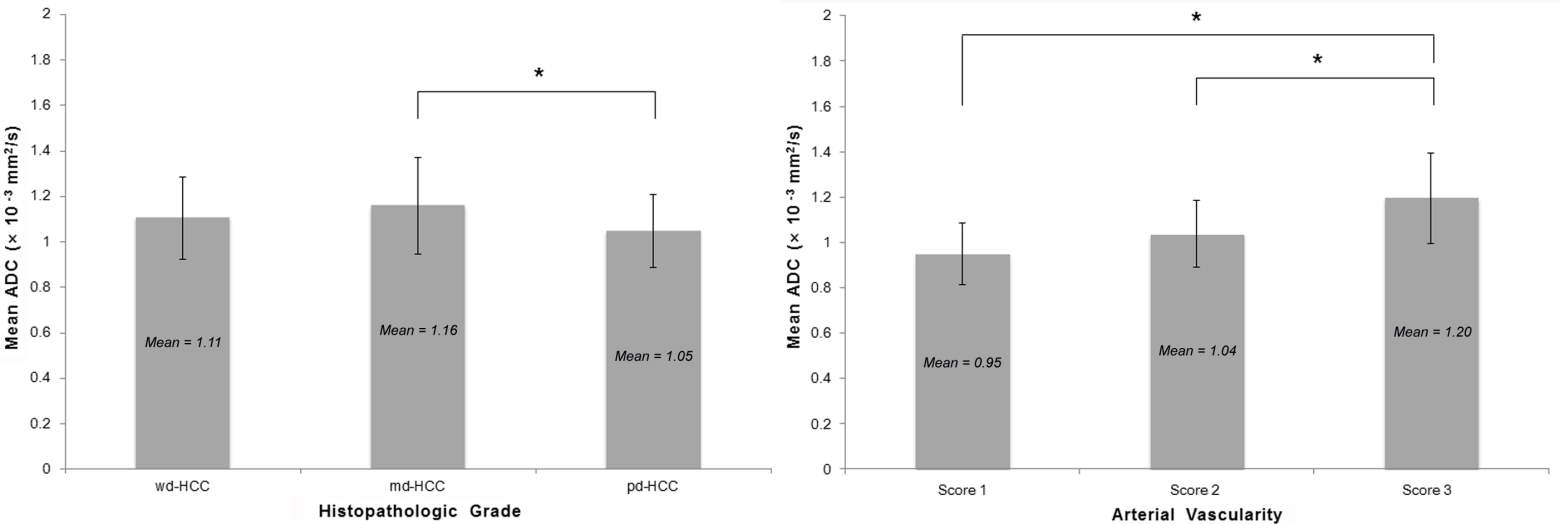
Relationship between apparent diffusion coefficients (ADCs) and histopathologic grades or arterial vascularity. For the histopathologic tumor grades (left), the ADC is significantly higher for md-HCC than for pd-HCC (asterisk, *P* = 0.010), while other comparisons show no significant differences. For the arterial vascularity of the tumors (right), the mean ADC is significantly higher for score 3 lesions (asterisks) than for score 1 lesions (*P* < 0.001) or score 2 lesions (*P* < 0.001). There is no significant difference in mean ADC values between score 1 and 2 lesions (*P* = 0.336). HCC, hepatocellular carcinoma; wd, well-differentiated; md, moderately-differentiated; pd, poorly-differentiated.

For the histopathologic tumor grades, the mean ADCs of wd-HCCs (1.11 ± 0.18 × 10^−3^mm^2^/s), md-HCCs (1.16 ± 0.21 × 10^−3^ mm^2^/s), and pd-HCCs (1.05 ± 0.16 × 10^−3^ mm^2^/s) showed overall different values (*P* = 0.013), but there was no trend of stratification (*P* = 0.323) among the three groups (Fig 3). On comparing the mean ADCs between two of the three histopathologic tumor grades, md-HCC showed a significantly higher ADC than pd-HCC (*P* = 0.010), while the other comparisons showed no significant differences (wd-HCC *vs*. md-HCC, *P* = 0.960; wd-HCC *vs*. pd-HCC, *P* = 0.968).

### ADC and arterial vascularities

The arterial vascularity of 15, 44, and 82 tumors was classified as score 1, 2, and 3 respectively; the mean ADC of HCCs increased significantly with increasing enhancement (*P* < 0.001) (Fig 3). The mean ADC of score 3 lesions (1.20 ± 0.20 × 10^−3^ mm^2^/s) was significantly higher than that of score 1 lesions (0.95 ± 0.14 × 10^−3^mm^2^/s; *P* < 0.001) or score 2 lesions (1.04 ± 0.15 × 10^−3^mm^2^/s; *P* < 0.001). The mean ADC value was not significantly different between score 1 and 2 lesions (*P* = 0.336).

### Subgroup analysis of tumor grades with arterial vascularities

For the relationship of ADCs in each histopathological tumor grade subgroup with arterial vascularity, score 3 lesions showed higher ADCs than score 1 lesions (*P* = 0.023, *P* < 0.001) or score 2 lesions (*P* = 0.021, *P* = 0.001) in the subgroups of wd-HCC and md-HCC, respectively. Only the score 3 lesions showed a statistically significant difference of mean ADCs in histopathologic tumor grade (md-HCC *vs*. pd-HCC, *P* = 0.017) (Tables 1 and 2).

**Table 1:** Mean apparent diffusion coefficient (×10^−3^mm^2^/s) in each subgroup of tumor grade and vascularity.

	Score 1	Score 2	Score 3	*P* value
**wd-HCCs**	0.898±0.025 (n = 2)	0.986±0.080 (n = 5)	1.218±0.156 (n = 9)	0.005
**md-HCCs**	0.946±0.149 (n = 8)	1.037±0.154 (n = 18)	1.228±0.202 (n = 57)	<0.001
**pd-HCCs**	0.978±0.150 (n = 5)	1.047±0.155 (n = 21)	1.072±0.175 (n = 16)	0.535
***P* value**	0.796	0.711	0.020	

Note.—Score 1, 2 and 3 mean non-hypervascular, slightly-hypervascular and markedly-hypervascular lesions, respectively. wd, well-differentiated; md, moderately-differentiated; pd, poorly-differentiated; HCC, hepatocellular carcinoma.

**Table 2:** *P* values of bonferroni correction for apparent diffusion coefficients in each subgroup of tumor grade and vascularity.

	*P* value
***Histopathologic Grade***	
**wd-HCCs**	
Score 1 vs Score 2	>0.999
Score 1 vs Score 3	0.023
Score 2 vs Score 3	0.021
**md-HCCs**	
Score 1 vs Score 2	0.787
Score 1 vs Score 3	<0.001
Score 2 vs Score 3	0.001
**pd-HCCs**	
Score 1 vs Score 2	>0.999
Score 1 vs Score 3	0.801
Score 2 vs Score 3	>0.999
***Arterial Vascularity***	
**Score 1**	
wd-HCCs vs md-HCCs	>0.999
wd-HCCs vs pd-HCCs	>0.999
md-HCCs vs pd-HCCs	>0.999
**Score 2**	
wd-HCCs vs md-HCCs	>0.999
wd-HCCs vs pd-HCCs	>0.999
md-HCCs vs pd-HCCs	>0.999
**Score 3**	
wd-HCCs vs md-HCCs	>0.999
wd-HCCs vs pd-HCCs	0.217
md-HCCs vs pd-HCCs	0.017

Note.—Score 1, 2 and 3 mean non-hypervascular, slightly-hypervascular and markedly-hypervascular lesions, respectively. wd, well-differentiated; md, moderately-differentiated; pd, poorly-differentiated; HCC, hepatocellular carcinoma.

## Discussion

Similar to earlier reports on genitourinary malignancy (*e*.*g*., bladder and cervix) using the ADC of DWI for tumor grading [28, 29], the potential use of ADC for depicting the histopathologic grade of HCC has also been suggested [20–27]. In the present study, however, there was no significant stratification of mean ADCs according to the tumor grades. The finding of comparable or lower ADCs of wd-HCCs, compared to those of md-HCCs, coincided with the prior results of Nasu et al [30], which showed that the histopathologic grade of HCC was not correlated with the ADC. As speculated [30], the cellular atypia of different nucleus-cytoplasm ratios, which primarily determined the histopathologic tumor grade, would be trivial in revealing gross differences in ADC on current DWI [31, 32].

Previous research on the correlation between arterial blood supply and grades of malignant hepatic nodules demonstrated that arterial blood supply increases in the early stage of HCC development and then decreases in the late stage [33]. Therefore, the ADC of HCC in DWI may reflect the degree of tumor vascularity rather than the histopathologic tumor grade, which supports the finding that md-HCCs showed higher ADC values than wd-HCCs and pd-HCCs because ADC is not only affected by molecular diffusion but also by microcapillary perfusion, especially by use of small *b* factors [34, 35]. Even when we used *b* = 50 instead of *b* = 0 for the ADC calculation to reduce the perfusion effect during DWI, the perfusion effect was still large enough to have a considerable influence on the ADCs in the present study.

**Fig 4:**
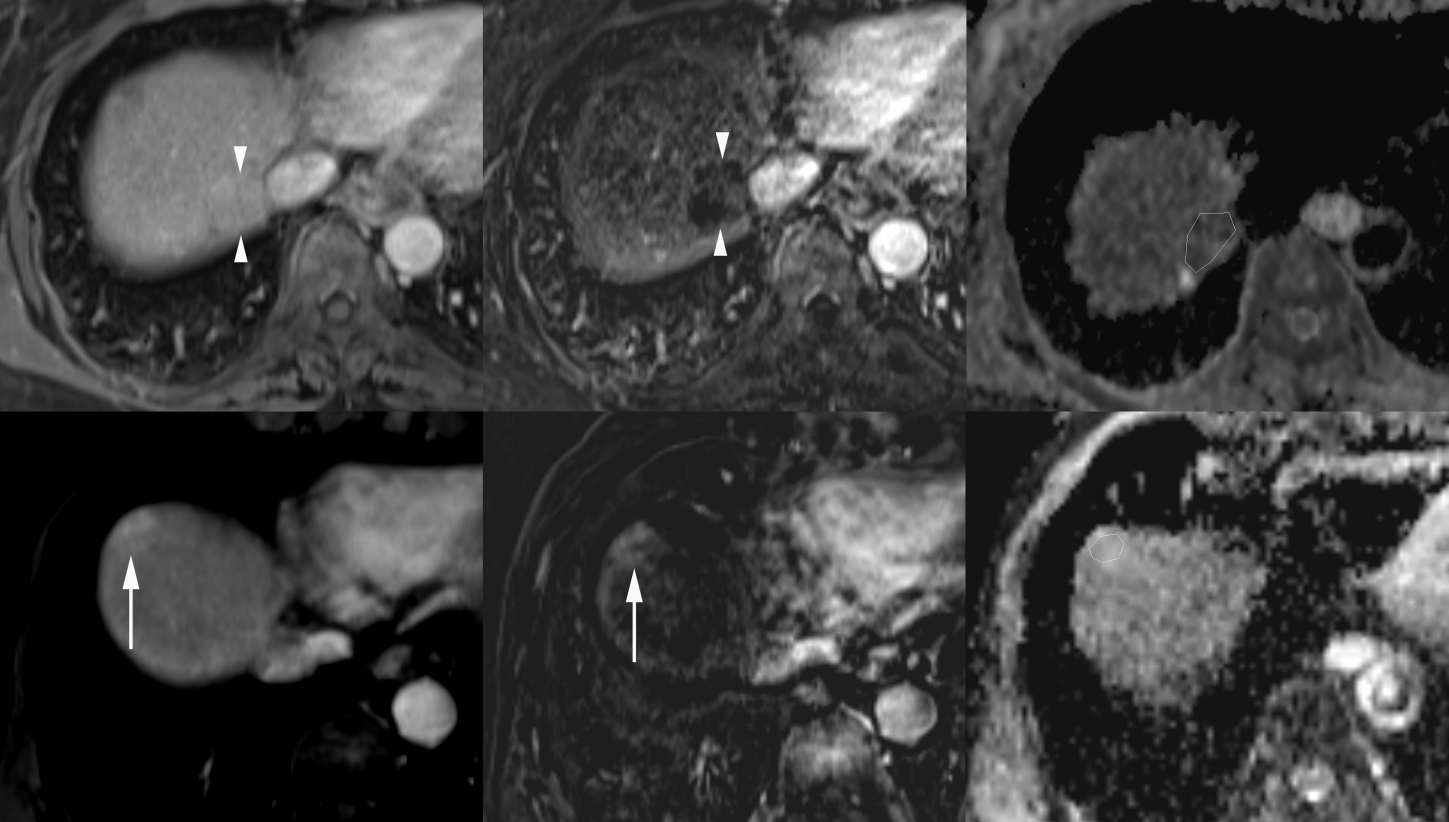
Diversity of tumor vascularity and apparent diffusion coefficients (ADCs) of well-differentiated hepatocellular carcinomas (wd-HCCs). A nonhypervascular wd-HCC on the arterial phase image (upper left) and the corresponding subtraction postcontrast image (upper middle) has a low ADC value (0.928 × 10^−3^ mm^2^/s) in the polygonal region of interest (ROI) on the ADC map (upper right). In another patient, a markedly hypervascular wd-HCC on the arterial phase image (lower left) and the corresponding subtraction postcontrast image (lower middle) shows a high ADC value (1.388 × 10^−3^ mm^2^/s) in the polygonal ROI on the ADC map (lower right).

Meanwhile, the discrepancies in the mean ADCs of wd-HCCs between the previous reports [20–27] and the present study may be explained by the inherent diversity of the imaging characteristics of wd-HCCs (Fig 4). In clinical practice, we have often experienced wd-HCCs showing either the typical appearance of classical HCC with hypervascularity and T2-weighted hyperintensity or the nonhypervascularity with T1-weighted iso-/hyper-intensity and T2-weighted iso-/hypo-intensity mimicking regenerating or dysplastic nodules. The fact that pathologists could use subjective criteria on a case-by-case basis in the differential diagnosis of borderline malignant cirrhotic nodules, including wd-HCC and dysplastic nodules, and in tumor grading may have also contributed to this contradiction [36].

Locating the ROI in the tumor and defining the representative tumor grades for rather heterogeneous tumor components may be debatable. Some investigators used minimal ADCs among the values measured on multiple ROIs in different locations of each tumor [22]. However, there was no significant difference between the minimal ADCs and average ADCs of the solid component as a representative value in a comparative analysis with histopathologic tumor grades [27]. If a necrotic or hemorrhagic tumor portion could be excluded from the ROI measurement, inadequate measurement or sampling error would be of no remarkable concern.

**Fig 5:**
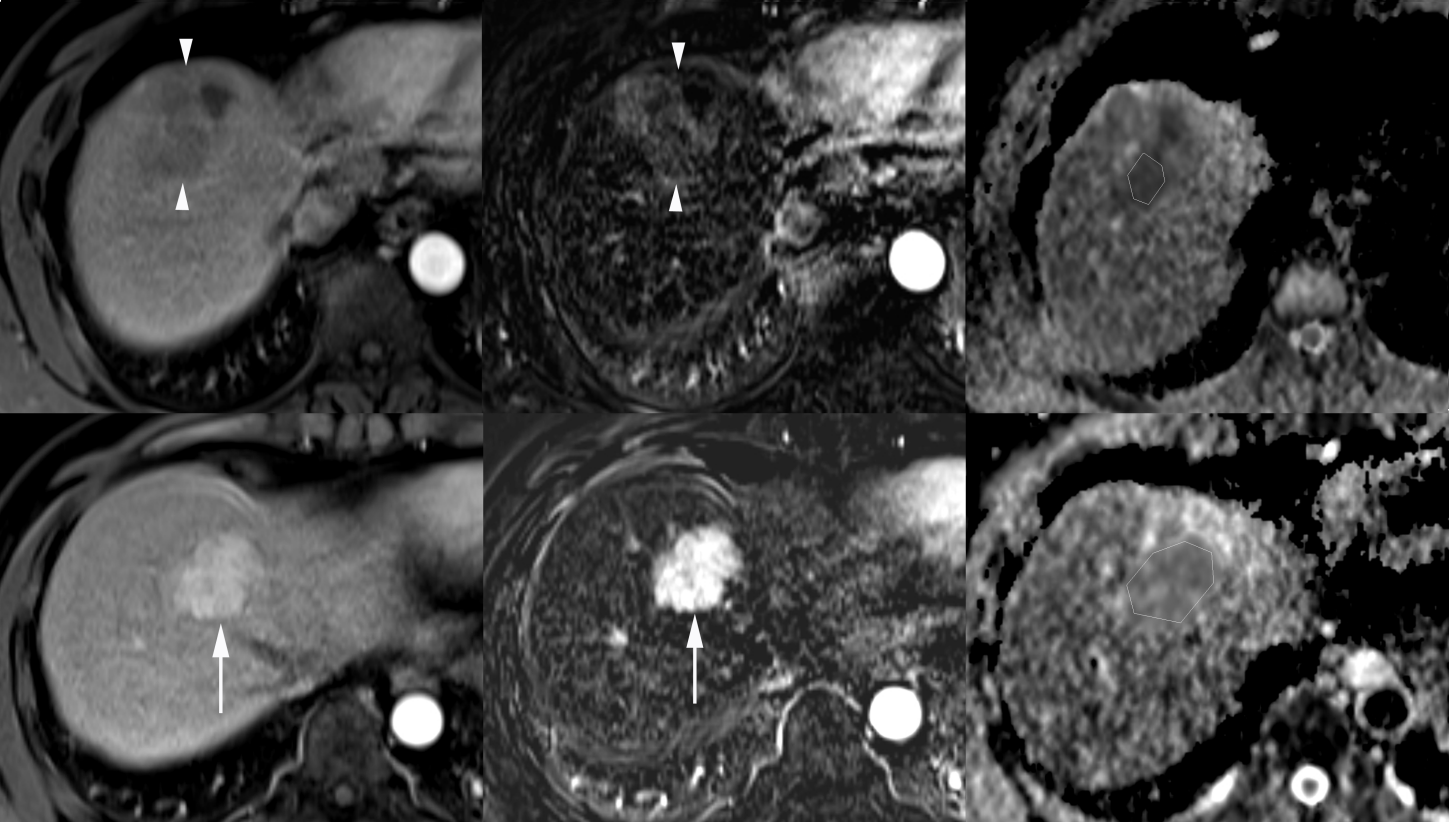
Constantly low apparent diffusion coefficients (ADCs) of poorly differentiated hepatocellular carcinomas (pd-HCCs). A nonhypervascular pd-HCC on the arterial phase image (upper left) and the corresponding subtraction postcontrast image (upper middle) shows a low ADC value (0.822 × 10^−3^ mm^2^/s) in the polygonal region of interest (ROI) on the ADC map (upper right). In another patient, a markedly hypervascular pd-HCC on the arterial phase image (lower left) and the corresponding subtraction postcontrast image (lower middle) also shows a low ADC value (0.896 × 10^−3^ mm^2^/s) in the polygonal ROI on the ADC map (lower right).

In the present study, the subgroup analysis of histopathologic tumor grades in each arterial vascularity group and vice versa revealed that ADC significantly increased with the arterial vascularity in the wd- and md-HCCs. However, the incremental degree was not sufficiently large to show statistical significance in the pd-HCCs (Fig 5). We did not have ADC data using *b* = 0, which could be more sensitive for perfusion fraction; these findings indicated that the different degrees of arterial vascularity were insufficient to grossly influence the ADC values in pd-HCCs. The structural atypia of high cellular density with distortion of extracellular stroma restricting the diffusion of water molecules in the intercellular spaces could be more influential than the degree of arterial vascularity in the pd-HCCs. Similar to prior reports [20–27], the consistently low ADC value in pd-HCCs may arise from the synergistic effect of low vascular perfusion and restricted water diffusion.

Recently, there have been several reports on the use of intravoxel incoherent motion (IVIM) imaging to determine the correlation of diffusion and perfusion parameters with histologic grades of HCC using a biexponential algorithm [37–39]. Despite prior studies suggesting the usefulness of IVIM for the prediction of histologic grades of HCC, there were inconsistencies in the findings of these studies [37–39]. A standardized protocol and a large study are needed to thoroughly test the suitability of IVIM for this purpose.

Our study has several limitations. First, arterial vascularity was not histologically assessed, and there were no real-time data of tumor perfusion. In a previous study involving IVIM, the subjectively determined arterial vascularity—on a three-grade scale similar to the one used in the present study—was well correlated with the perfusion fraction determined on IVIM [37]. Based on the results of another study showing the relationship of the arterial enhancement during dynamic imaging with tumor vascularity [40], we subjectively determined the arterial vascularity in the present study. Despite a combined review of dynamic images with corresponding subtraction images and good interobserver agreement, more standardized quantitative data of continuous variables for perfusion are needed to reinforce the results of the present study. Second, the major histopathologic tumor grade was used for the analysis in the present study. However, the patients’ prognosis depended on the worst grade in the HCCs; in the preliminary analysis, however, the worst tumor grades did not show any statistically significant results. This finding may indicate limited value of DWI as a biomarker for predicting the tumor prognosis of HCCs.

In conclusion, the ADC of HCCs could not stratify the histopathologic tumor grades, probably because of the variable ADC and arterial vascularity of wd-HCCs and the insufficient difference of diffusivity between the wd- and md-HCCs. The degree of arterial phase enhancement might be rather well correlated with the ADC values for wd-HCCs and md-HCCs while pd-HCCs show constantly low ADCs regardless of arterial vascularities. Based on the results of the present study, hypervascular HCCs with low ADC could be interpreted as pd-HCCs with poor prognosis, while it remains difficult to differentiate pd-HCCs from wd-HCCs for nonhypervascular lesions using only the ADC values. Due to the limited ability to stratify the histopathologic tumor grades, the ADC should be used with caution as a tumor biomarker in determining the prognosis of HCCs.

## References

[1] Torre LA, Bray F, Siegel RL, Ferlay J, Lortet-Tieulent J, Jemal A. Global cancer statistics, 2012. CA Cancer J Clin 2015;65:87–108. doi: 10.3322/caac.21262 25651787 10.3322/caac.21262

[2] Jonas S, Bechstein WO, Steinmuller T, Herrmann M, Radke C, Berg T, et al Vascular invasion and histopathologic grading determine outcome after liver transplantation for hepatocellular carcinoma in cirrhosis. Hepatology 2011;33:1080–1086. 10.1053/jhep.2001.23561 11343235

[3] Perez-Saborido B, de los Galanes SJ, Meneu-Diaz JC, Romero CJ, Elola-Olaso AM, Suarez YF, et al Tumor recurrence after liver transplantation for hepatocellular carcinoma: recurrence pathway and prognostic factors. Transplant Proc 2007;39:2304–2307. doi: 10.1016/j.transproceed.2007.06.059 17889172 10.1016/j.transproceed.2007.06.059

[4] Haratake J, Takeda S, Kasai T, Nakano S, Tokui N. Predictable factors for estimating prognosis of patients after resection of hepatocellular carcinoma. Cancer 1993;72:1178–1183. 7687921 10.1002/1097-0142(19930815)72:4<1178::aid-cncr2820720408>3.0.co;2-q

[5] Fitzmorris P, Shoreibah M, Anand BS, Singal AK. Management of hepatocellular carcinoma. J Cancer Res Clin Oncol 2015;141:861–876. doi: 10.1007/s00432-014-1806-0 25158999 10.1007/s00432-014-1806-0 PMC11823677

[6] Zimmerman MA, Ghobrial RM, Tong MJ, Hiatt JR, Cameron AM, Hong J, et al Recurrence of hepatocellular carcinoma following liver transplantation: a review of preoperative and postoperative prognostic indicators. Arch Surg 2008;143:182–188. doi: 10.1001/archsurg.2007.39 18283144 10.1001/archsurg.2007.39

[7] Suh KS, Cho EH, Lee HW, Shin WY, Yi NJ, Lee KU. Liver transplantation for hepatocellular carcinoma in patients who do not meet the Milan criteria. Dig Dis 2007; 25:329–333. doi: 10.1159/000106913 17960068 10.1159/000106913

[8] Cillo U, Vitale A, Bassanello M, Brolese A, Vitale A, Boccagni P, et al Liver transplantation for the treatment of moderately or well-differentiated hepatocellular carcinoma. Ann Surg 2004;239:150–159. doi: 10.1097/01.sla.0000109146.72827.76 14745321 10.1097/01.sla.0000109146.72827.76 PMC1356206

[9] Jensen JH, Helpern JA, Ramani A, Lu H, Kaczynski K. Diffusional kurtosis imaging: the quantification of non-gaussian water diffusion by means of magnetic resonance imaging. Magn Reson Med 2005;53:1432–1440. doi: 10.1002/mrm.20508 15906300 10.1002/mrm.20508

[10] Kele PG, van der Jagt EJ. Diffusion weighted imaging in the liver. World J Gastroenterol 2010;16:1567–1576. doi: 10.3748/wjg.v16.i13.1567 20355235 10.3748/wjg.v16.i13.1567 PMC2848365

[11] Kim HJ, Lee SS, Byun JH, Kim JC, Yu CS, Park SH, et al Incremental value of liver MR imaging in patients with potentially curable colorectal hepatic metastasis detected at CT: A prospective comparison of diffusion-weighted imaging, Gadoxetic acid-enhanced MR imaging, and a combination of both MR techniques. Radiology 2015;274:712–722. doi: 10.1148/radiol.14140390 25286324 10.1148/radiol.14140390

[12] Nasu K, Kuroki Y, Nawano S, Kuroki S, Tsukamoto T, Yamamoto S, et al Hepatic metastases: diffusion-weighted sensitivity-encoding versus SPIO-enhanced MR imaging. Radiology 2006;239:122–130. doi: 10.1148/radiol.2383041384 16493012 10.1148/radiol.2383041384

[13] Parikh T, Drew SJ, Lee VS, Wong S, Hecht EM, Babb JS, et al Focal liver lesion detection and characterization with diffusion-weighted MR imaging: comparison with standard breast-hold T2-weighted imaging. Radiology 2008;246:812–822. doi: 10.1148/radiol.2463070432 18223123 10.1148/radiol.2463070432

[14] Lowenthal D, Zeile M, Lim WY, Wybranski C, Fischbach F, Wieners G, et al Detection and characterisation of focal liver lesions in colorectal carcinoma patients: comparison of diffusion-weighted and Gd-EOB-DTPA enhanced MR imaging. Eur Radiol 2011;21:832–840. doi: 10.1007/s00330-010-1977-2 20886339 10.1007/s00330-010-1977-2

[15] Kim YK, Kim CS, Han YM, Lee YH. Detection of liver malignancy with gadoxetic acid-enhanced MRI: is addition of diffusion-weighted MRI beneficial? Clin Radiol 2011;66:489–496. doi: 10.1016/j.crad.2010.09.007 21367403 10.1016/j.crad.2010.09.007

[16] Kenis C, Deckers F, De Foer B, Van Mieghem F, Van Laere S, Pouillon M. Diagnosis of liver metastases: can diffusion-weighted imaging (DWI) be used as a stand alone sequence? Eur J Radiol 2012;81:1016–1023. doi: 10.1016/j.ejrad.2011.02.019 21377305 10.1016/j.ejrad.2011.02.019

[17] Partridge SC, Mullins CD, Kurland BF, Allain MD, DeMartini WB, Eby PR, et al Apparent diffusion coefficient values for discriminating benign and malignant breast MRI lesions: effects of lesion type and size. AJR Am J Roentgenol 2010;194:1664–1673. doi: 10.2214/AJR.09.3534 20489111 10.2214/AJR.09.3534

[18] Lee MH, Kim SH, Park MJ, Park CK, Rhim H. Gadoxetic acid-enhanced hepatobiliary phase MRI and high-b-value diffusion-weighted imaging to distinguish well-differentiated hepatocellular carcinomas from benign nodules in patients with chronic liver disease. AJR Am J Roentgenol 2011;197:W868–875. doi: 10.2214/AJR.10.6237 22021534 10.2214/AJR.10.6237

[19] Inchingolo R, De Gaetano AM, Curione D, Ciresa M, Miele L, Pompili M, et al Role of diffusion-weighted imaging, apparent diffusion coefficient and correlation with hepatobiliary phase findings in the differentiation of hepatocellular carcinoma from dysplatic nodules in cirrhotic liver. Eur Radiol 2015;25:1087–1096. doi: 10.1007/s00330-014-3500-7 25430005 10.1007/s00330-014-3500-7

[20] Muhi A, Ichikawa T, Motosugi U, Sano K, Matsuda M, Kitamura T, et al High-b-value diffusion-weighted MR imaging of hepatocellular lesions: estimation of grade of malignancy of hepatocellular carcinoma. J Magn Reson Imaging 2009;30:1005–1011. doi: 10.1002/jmri.21931 19856432 10.1002/jmri.21931

[21] Heo SH, Jeong YY, Shin SS, Kim JW, Lim HS, Lee JH, et al Apparent diffusion coefficient value of diffusion-weighted imaging for hepatocellular carcinoma: correlation with the histologic differentiation and the expression of vascular endothelial growth factor. Korean J Radiol 2010;11:295–303. doi: 10.3348/kjr.2010.11.3.295 20461183 10.3348/kjr.2010.11.3.295 PMC2864856

[22] Nishie A, Tajima T, Asayama Y, Ishigami K, Kakihara D, Nakayama T, et al Diagnostic performance of apparent diffusion coefficient for predicting histological grade of hepatocellular carcinoma. Eur J Radiol 2011;80:e29–33. doi: 10.1016/j.ejrad.2010.06.019 20619566 10.1016/j.ejrad.2010.06.019

[23] Chang WC, Chen RC, Chou CT, Lin CY, Yu CY, Liu CH, et al Histological grade of hepatocellular carcinoma correlates with arterial enhancement on gadoxetic acid-enhanced and diffusion-weighted MR images. Abdom Imaging 2014;39:1202–1212. doi: 10.1007/s00261-014-0168-z 24869790 10.1007/s00261-014-0168-z

[24] Chen J, Wu M, Liu R, Li S, Gao R, Song B. Preoperative evaluation of the histological grade of hepatocellular carcinoma with diffusion-weighted imaging: a meta-analysis. PLoS One 2015;10:e0117661 doi: 10.1371/journal.pone.0117661 25658359 10.1371/journal.pone.0117661 PMC4320049

[25] Guo W, Zhao S, Yang Y, Shao G. Histological grade of hepatocellular carcinoma predicted by quantitative diffusion-weighted imaging. Int J Clin Exp Med 2015;8:4164–4169. 26064326 PMC4443160

[26] Tang Y, Wang H, Ma L, Zhang X, Yu G, Li J, et al Diffusion-weighted imaging of hepatocellular carcinomas: a retrospective analysis of correlation between apparent diffusion coefficients and histological grade. Abdom Radiol (NY) 2016;41:1539–1545. 27003574 10.1007/s00261-016-0715-x

[27] Li X, Zhang K, Shi Y, Wang F, Meng X. Correlations between the minimum and mean apparent diffusion coefficient values of hepatocellular carcinoma and tumor grade. J Magn Reson Imaging 2016;44:1442–1447. doi: 10.1002/jmri.25323 27228086 10.1002/jmri.25323

[28] Takeuchi M, Sasaki S, Ito M, Okada S, Takahashi S, Kawai T, et al Urinary bladder cancer: diffusion-weighted MR imaging—accuracy for diagnosing T stage and estimating histologic grade. Radiology 2009; 251:112–121. doi: 10.1148/radiol.2511080873 19332849 10.1148/radiol.2511080873

[29] Tamai K, Koyama T, Saga T, Umeoka S, Mikami Y, Fujii S, et al Diffusion-weighted MR imaging of uterine endometrial cancer. J Magn Reson Imaging 2007;26:682–687. doi: 10.1002/jmri.20997 17729360 10.1002/jmri.20997

[30] Nasu K, Kuroki Y, Tsukamoto T, Nakajima H, Mori K, Minami M. Diffusion-weighted imaging of surgically resected hepatocellular carcinoma: imaging characteristics and relationship among signal intensity, apparent diffusion coefficient, and histopathologic grade. AJR Am J Roentgenol 2009;193:438–444. doi: 10.2214/AJR.08.1424 19620441 10.2214/AJR.08.1424

[31] White NS, Dale AM. Distinct effects of nuclear volume fraction and cell diameter on high b-value diffusion MRI contrast in tumors. Magn Reson Med 2014;72:1435–1443. doi: 10.1002/mrm.25039 24357182 10.1002/mrm.25039 PMC5400014

[32] Xu J, Does MD, Gore JC. Sensitivity of MR diffusion measurements to variations in intracellular structure: effects of nuclear size. Magn Reson Med 2009;61:828–833. doi: 10.1002/mrm.21793 19205020 10.1002/mrm.21793 PMC2749035

[33] Asayama Y, Yoshimitsu K, Nishihara Y, Irie H, Aishima S, Taketomi A, et al Arterial blood supply of hepatocellular carcinoma and histologic grading: radiologic-pathologic correlation. AJR Am J Roentgenol 2008;190:W28–34. doi: 10.2214/AJR.07.2117 18094269 10.2214/AJR.07.2117

[34] Bruegel M, Holzapfel K, Gaa J, Woertler K, Waldt S, Kiefer B, et al Characterization of focal liver lesions by ADC measurements using a respiratory triggered diffusion-weighted single-shot echo-planar MR imaging technique. Eur Radiol 2008;18:477–485. doi: 10.1007/s00330-007-0785-9 17960390 10.1007/s00330-007-0785-9

[35] Gourtsoyianni S, Papanikolaou N, Yarmenitis S, Maris T, Karantanas A, Gourtsoyiannis N. Respiratory gated diffusion-weighted imaging of the liver: value of apparent diffusion coefficient measurements in the differentiation between most commonly encountered benign and malignant focal liver lesions. Eur Radiol 2008;18:486–492. doi: 10.1007/s00330-007-0798-4 17994317 10.1007/s00330-007-0798-4

[36] Kojiro M. Diagnostic discrepancy of early hepatocellular carcinoma between Japan and West. Hepatol Res 2007;37 Suppl 2:S121–124. 17877472 10.1111/j.1872-034X.2007.00174.x

[37] Woo S, Lee JM, Yoon JH, Joo I, Han JK, Choi BI. Intravoxel incoherent motion diffusion-weighted MR imaging of hepatocellular carcinoma: correlation with enhancement degree and histologic grade. Radiology 2014;270:758–767. doi: 10.1148/radiol.13130444 24475811 10.1148/radiol.13130444

[38] Granata V, Fusco R, Catalano O, Guarino B, Granata F, Tatangelo F, et al Intravoxel incoherent motion (IVIM) in diffusion-weighted imaging (DWI) for hepatocellular carcinoma: correlation with histologic grade. Oncotarget 2016;7:79357–79364. doi: 10.18632/oncotarget.12689 27764817 10.18632/oncotarget.12689 PMC5346719

[39] Shan Q, Chen J, Zhang T, Yan R, Wu J, Shu Y, et al Evaluating histologic differentiation of hepatitis B virus-related hepatocellular carcinoma using intravoxel incoherent motion and AFP levels alone and in combination. Abdom Radiol (NY) 2017;42:2079–2088. 28337521 10.1007/s00261-017-1107-6

[40] Kim CK, Lim JH, Park CK, Choi D, Lim HK, Lee WJ. Neoangiogenesis and sinusoidal capillarization in hepatocellular carcinoma: correlation between dynamic CT and density of tumor microvessels. Radiology 2005;237: 529–534. doi: 10.1148/radiol.2372041634 16244261 10.1148/radiol.2372041634

